# Detection of potentially pathogenic enteric viruses in environmental samples from Kenya using the bag-mediated filtration system

**DOI:** 10.2166/ws.2019.046

**Published:** 2019-03-12

**Authors:** Walda B. van Zyl, Nicolette A. Zhou,, Marianne Wolfaardt, Peter N. Matsapola, Fhatuwani B. Ngwana, Erin M. Symonds, Christine S. Fagnant-Sperati, Jeffry H. Shirai, Alexandra L. Kossik, Nicola K. Beck, Evans Komen, Benlick Mwangi, James Nyangao, David S. Boyle, Peter Borus, Maureen B. Taylor, J. Scott Meschke

**Affiliations:** 1 **Walda B. van Zyl, Marianne Wolfaardt, Peter N. Matsapola, Fhatuwani B. Ngwana, Maureen B. Taylor** Department of Medical Virology, University of Pretoria, Faculty of Health Sciences, Private Bag X323, Arcadia 0007, South Africa; 2 **Nicolette A. Zhou, Christine S. Fagnant-Sperati, Jeffry H. Shirai, Alexandra L. Kossik, Nicola K. Beck, J. Scott Meschke** (corresponding author) Department of Environmental and Occupational Health Sciences, University of Washington, 4225 Roosevelt Way NE, Suite 100, Seattle, WA 98105, USA; 3 **Erin M. Symonds** College of Marine Science, University of South Florida, 830 1st St S, St Petersburg, FL 33701, USA; 4 **Evans Komen, Benlick Mwangi, James Nyangao, Peter Borus** Centre for Viral Research, Kenya Medical Research Institute, Mbagathi Road, P.O. Box 54628, Nairobi 00200, Kenya; 5 **David S. Boyle** PATH, 2201 Westlake Ave, Suite 200, Seattle, WA 98121, USA

**Keywords:** BMFS, enteric viruses, environmental monitoring, environmental surveillance, pepper mild mottle virus, wastewater

## Abstract

Enteric virus environmental surveillance via a highly sensitive method is critical, as many enteric viruses have low infectious doses and can persist in the environment for extended periods. This study determined the potential of the novel bag-mediated filtration system (BMFS) to recover human enteric viruses and pepper mild mottle virus (PMMoV) from wastewater and wastewater-impacted surface waters, examined PMMoV use as a fecal contamination indicator in Kenya, and identified potential BMFS process controls. From April 2015 to April 2016, BMFS samples were collected from seven sites in Kenya (*n* = 59). Enteroviruses and PMMoV were detected in 100% of samples, and human adenovirus, human astrovirus, hepatitis A virus, norovirus GI, norovirus GII, sapovirus, and human rotavirus were detected in the majority of samples. The consistent detection of enteroviruses and PMMoV suggests that these viruses could be used as indicators in similarly fecally contaminated sites and BMFS process controls. As contamination of surface water sources remains a global issue, enteric virus environmental surveillance is necessary. This study demonstrates an effective way to sample large volumes of wastewater and wastewater-impacted surface waters for the detection of multiple enteric viruses simultaneously.

## INTRODUCTION

Waterborne diseases are responsible for approximately 3.4 million deaths per year globally, and enteric viruses are frequently implicated in waterborne disease outbreaks (Gibson 2014). Enteric virus shedding in stool from infected individuals can contaminate environmental waters, leading to waterborne outbreaks (Gibson 2014). Enteric viruses can be detected in wastewater and wastewater-impacted surface waters in areas with poor sanitation (Gibson *et al*. 2011; Masachessi et al. 2018). Many viruses have low infectious doses and can persist in the environment for extended periods (Prevost *et al*. 2016). Environmental surveillance informs on virus prevalence in the community, and is a critical adjunct method as clinical surveillance is limited to health-seeking, symptomatic individuals. Environmental surveillance can detect silently circulating pathogens, which helps guide vaccine efforts (World Health Organization (WHO) 2015). Therefore, environmental surveillance is a vital supplement to clinical surveillance for informing on disease burdens within a community (WHO 2015).

To determine if an environmental surveillance site is fecally contaminated, indicators have been identified, including *Escherichia coli, Enterococci,* somatic coliphages (Ashbolt et al. 2001), enterovirus (EV) (Symonds et al. 2009), and recently pepper mild mottle virus (PMMoV) (Rosario et al. 2009). PMMoV is a non-enveloped, positivesense single-stranded RNA virus in the *Tobamovirus* genus (King *et al*. 2011) and is of dietary origin (Rosario *et al*. 2009). PMMoV is of interest as an indicator species during environmental surveillance as it closely correlates with enteric viruses (Hamza*et al*. 2011; Symonds *et al*. 2018), and is consistently detected in domestic wastewater at concentrations equal to or greater than human enteric viruses (Kitajima *et al*. 2014). While PMMoV has been found at low concentrations in fecal samples from chickens, cows, seagulls, and geese (Rosario *et al*. 2009; Hamza *et al*. 2011), its frequently high concentrations in domestic wastewater makes it a useful indicator and enteric virus index virus (Symonds *et al*. 2018). PMMoV has been used globally as a fecal contamination indicator in source waters; however, its presence in African waters has not yet been examined (Symonds *et al*. 2018).

A novel environmental surveillance method called the bag-mediated filtration system (BMFS) was developed for detection of poliovirus, an enteric virus, from environmental waters and first tested in Kenya (Fagnant *et al*. 2018). The BMFS enables in-field collection and filtration of large sample volumes (3–6 L wastewater or wastewater-impacted waters) (Fagnant *et al*. 2018; Zhou *et al*. 2018). Filters are further processed in the laboratory, including filter elution and secondary concentration (Fagnant *et al*. 2018). BMFS advantages include the ability to ship compact cartridge filters rather than large-volume, potentially hazardous water samples, and capacity to process large sample volumes for a high effective volume assayed. The BMFS results in improved sensitivity for poliovirus surveillance compared to the gold standard method that processes 500-mL by aqueous two-phase separation (Zhou *et al*. 2018). This study examines the ability of the BMFS to sample and concentrate a diverse array of enteric viruses simultaneously.

The objective of this study was to (1) determine the potential for the BMFS to recover diverse domestic wastewater-related viruses from wastewater and wastewaterimpacted surface waters, (2) determine the potential to use PMMoV as a fecal contamination indicator in Kenya, and (3) identify potential BMFS process controls. BMFS environmental samples from seven Kenyan sites were analyzed for EV, human adenovirus (HAdV), human astrovirus (HAstV), hepatitis A virus (HAV), norovirus GI (NoV GI), norovirus GII (NoV GII), sapovirus (SaV), human rotavirus (HRV), and PMMoV.

### METHODS

#### Study design

Fecally polluted water samples were collected from 14 April 2015 to 16 April 2016 at seven sites in Kenya (*n* = 59; Supplementary Material, available with the online version of this paper). During the first 14 sampling events, two samples were collected sequentially (<5 minutes) at the same site. Single samples were collected during the last 31 sampling events, resulting in 59 samples.

#### Sample collection and processing

BMFS samples were collected and filtered as described previously (Fagnant *et al*. 2018; Zhou *et al*. 2018). The average volume filtered was 2.9 ± 0.1 L (95% confidence intervals [CI]; *n* = 57). Filters were shipped to the University of Pretoria (UP) for elution and secondary concentration as previously described (Fagnant *et al*. 2018) (Supplementary Material).

#### Nucleic acid extraction

At UP, secondary concentrate aliquots were chloroform extracted, followed by addition of 5 × 10^4^ copies of mengovirus (extraction control) and nucleic acid extraction. The semi-automated NucliSENS^®^ easyMAG^®^ instrument and accessory products (bioMérieux SA, Marcy-I’Étoile, France) were used according to manufacturer’s instructions, with a 1,000-μL input volume and nucleic acid elution in 100-μL.

BMFS secondary concentrate aliquots were shipped to United States Centers for Disease Control and Prevention where they were processed for PMMoV analysis. Samples were chloroform extracted and RNA extraction was performed using QIAamp^®^ Viral RNA Kits (Qiagen, Hilden, Germany) according to manufacturer’s instructions, with an increase in input volume to 280-μL, and RNA elution in 60-μL. RNA extracts were shipped to the University of Washington (UW) for PMMoV analysis.

#### RT-PCR

At UP, samples were analyzed for EV, HAstV, HAV, NoV GI, NoV GII, and HRV by direct real-time RT-PCR using CeeramTools^®^ kits (bioMérieux) according to manufacturer’s instructions. In-house real-time PCR assays for HAdV were conducted using TaqMan^®^ Environmental Master Mix 2.0 (Thermo Fisher Scientific Inc., Waltham, MA, USA) and published primers (Heim *et al*. 2003), and for SaV using QuantiTect^®^ Probe RT-PCR Kits (Qiagen, Hilden, Germany) and published primers (Murray *et al*. 2013). At UW, samples were analyzed for PMMoV by direct RT-qPCR using a published assay (Symonds *et al*. 2016) (Supplementary Material, Table S1, available online).

#### Analyses

Variability in Cq values over time were analyzed using Shewhart control charts (Microsoft Excel 2016). Upper and lower warning levels equaled average ± one standard deviation. Upper and lower control levels equaled average ± three standard deviations.

Difference among groups of data were tested using analysis of variance (ANOVA) with a post-hoc Tukey Honest Significant Difference Test (RStudio version 1.1.423).

### RESULTS AND DISCUSSION

#### Sequentially collected BMFS samples

Replicate samples were collected during 14 sampling events. For these replicates, in one instance a virus was identified in only one replicate (HAstV on 13 May 2015 from Eastleigh B). Cq values were used to estimate concentration of viruses in replicate filters (Table S2, available with the online version of this paper). Typically, Cq values were within 2 Cq for replicate filters (87.3%). For EV, HAV, and PMMoV, Cq values were within 2 Cq for all replicate filters. For NoV GII and SaV, Cq values were within 2 Cq for all except one replicate filter set. Results were within 2 Cq for all except three replicate filter sets for HAdV, NoV GI, and HRV and for all except six replicate filter sets for HAstV (including the non-detect in one filter on 13 May 2015). Of the eight sampling events resulting in a 2+ Cq difference between replicate filters, five of these events (62.5%) had two or more viruses with a 2+ Cq difference. While replicate samples were collected as close together as possible, differences in virus concentrations may exist between collected samples. Additionally, for replicate samples, slightly different volumes were filtered, particularly for samples from Eastleigh B on 13 May 2015 (2.5-L and 3-L) and Eastleigh A on 26 May 2015 (2.5-L and 3-L). These factors may contribute to the discordance in replicate sample Cq values.

#### Enteric virus detection

Diverse enteric viruses were frequently detected in BMFS samples, with detection in 88–100% of samples, depending on the virus (Table 1). When considering all sites and samples, EVs were present at high concentrations (i.e., low Cq; average Cq of 25.7), with little variability (95% CI of 0.4) (Table 2). EVs were detected with lower Cq values when compared to other enteric viruses.

**Table 1:** Frequency of viral detection in BMFS samples from multiple Kenyan sites

Site	EV	HAdV	HAstV	HAV	NoV GI	NoV GII	SaV	HRV	PMMoV	n
Nairobi Kibera	100%	100%	100%	100%	93%	86%	100%	100%	100%	14
Starehe	100%	100%	100%	100%	100%	100%	100%	93%	100%	14
Eastleigh A	100%	100%	100%	100%	92%	92%	83%	75%	100%	12
Eastleigh B	100%	100%	92%	100%	100%	100%	100%	83%	100%	12
Mombasa										
Kipevu	100%	100%	100%	100%	100%	100%	100%	100%	100%	3
Garissa										
Bullah Sheikh	100%	100%	100%	100%	100%	100%	100%	67%	100%	3
Kisumu										
Kisumu Polytechnic	100%	100%	100%	100%	100%	100%	100%	100%	100%	1
Average of all sites	100%	100%	98%	100%	97%	95%	97%	88%	100%	59

BMFS, bag-mediated filtration system; EV, enterovirus; HAdV, human adenovirus; HAstV, human astrovirus; HAV, hepatitis A virus; NoV GI, norovirus GI; NoV GII, norovirus GII; SaV, sapovirus; HRV, human rotavirus; PMMoV, pepper mild mottle virus.

**Table 2:** Real-time RT-PCR results, reported as the average quantification cycle, from the direct analysis of the BMFS samples collected from multiple Kenyan sites

	Cq ±95% CI							
Site	EV	HAdV	HAstV	HAV	NoV GI	NoV GII	SaV	HRV
Nairobi Kibera	25.7 ± 1.2	30.5 ± 1.8	29.7 ± 2.5	29.6 ± 1.7	32.2 ± 2.0	32.2 ± 1.8	32.3 ± 2.2	27.4 ± 2.5
Starehe	25.2 ± 0.8	29.2 ± 1.0	30.2 ± 1.7	27.6 ± 1.1	32.4 ± 1.5	31.9 ± 1.8	31.7 ± 1.3	29.3 ± 2.0
Eastleigh A	26.0 ± 0.7	30.1 ± 3.3	32.8 ± 1.6	31.9 ± 1.8	32.2 ± 1.8	32.0 ± 2.2	32.3 ± 1.8	30.8 ± 0.9
Eastleigh B	25.3 ± 0.7	32.2 ± 0.9	30.8 ± 2.6	31.0 ± 1.7	32.6 ± 1.5	30.7 ± 2.0	33.6 ± 1.7	31.4 ± 2.0
Mombasa								
Kipevu	25.3 ± 4.3	30.5 ± 3.5	28.3 ± 7.6	29.7 ± 7.1	34.7 ± 15.6	29.7 ± 13.9	32.1 ± 6.8	27.8 ± 7.6
Garissa								
Bullah Sheikh	27.7 ± 3.9	32.2 ± 4.0	32.4 ± 7.2	32.2 ± 7.4	33.9 ± 8.4	31.0 ± 14.8	34.9 ± 6.4	25.4 ± 2.0
Kisumu								
Kisumu Polytechnic	27.27	26.25	34.07	29.93	32.37	28.73	32.96	29.77
Average of all sites	25.7 ± 0.4	30.4 ± 0.8	30.8 ± 0.9	30.0 ± 0.8	32.5 ± 0.8	31.5 ± 0.9	32.6 ± 0.8	29.2 ± 1.0

RT-PCR, reverse transcription polymerase chain reaction; BMFS, bag-mediated filtration system; Cq, quantification cycle, where low Cq values reflects high virus concentrations, and high Cq values reflects low virus concentrations; CI, confidence interval; EV, enterovirus; HAdV, human adenovirus; HAstV, human astrovirus; HAV, hepatitis A virus; NoV GI, norovirus GI; NoV GII, norovirus GII; SaV, sapovirus; HRV, human rotavirus.

**Table 3:** qRT-PCR results for detection of PMMoV, reported as the average quantification cycle, from the direct analysis of BMFS samples collected from multiple Kenyan sites

Site	PMMoV Cq ±95% CI	PMMoVgene copies per reaction ±95% CI	Estimated gene copies L^1^ sample	n
Nairobi				
Kibera	28.1 ± 1.5	478 ± 225	4.42 × 10^5^	14
Starehe	26.0 ± 1.1	1,085 ± 311	1.00 × 10^6^	14
Eastleigh A	27.3 ± 1.6	611 ± 234	5.64 × 10^5^	12
Eastleigh B				
Mombasa	27.4 ± 0.8	397 ± 93	3.67 × 10^5^	12
Kipevu				
Garissa	27.2 ± 6.3	1,100 ± 1,279	1.02 × 10^6^	3
Bullah Sheikh				
Kisumu	28.2 ± 3.6	469 ± 346	4.33 × 10^5^	3
Kisumu Polytechnic	30.0	146	1.35 × 10^5^	1
Average of all sites	27.3 ± 0.6	658 ± 120	6.08 × 10^5^	59

RT-PCR, reverse transcription quantitative polymerase chain reaction; PMMoV, pepper mild mottle virus; BMFS, bag-mediated filtration system; Cq, quantification cycle, where low Cq values reflects high virus concentrations, and high Cq values reflects low virus concentrations; CI, confidence intervals.

**Figure 1:**
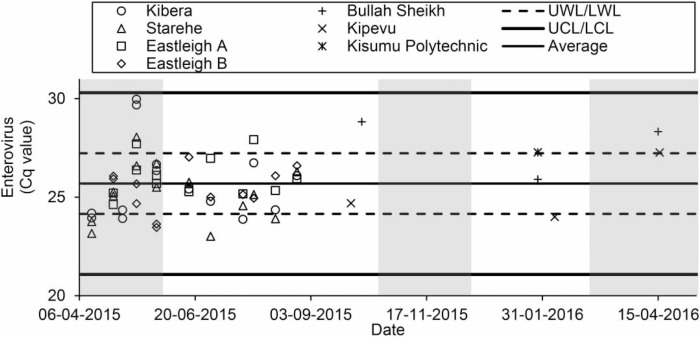
Detection of enterovirus. Shaded areas show the rainy season. UWL and LWL are the upper and lower warning levels (average ± one standard deviation). UCL and LCL are the upper and lower control levels (average ± three standard deviation).

A large variability in Cq values (>8) over time was seen for all human enteric viruses, with the exception of EVs (Figure 1 and Figures S1–S7, available online). This was most pronounced for Kibera samples. For human enteric viruses, Cq values were within the upper and lower warning levels for 58.6 to 67.9% of samples, outside the upper and lower warning levels for 32.1 to 41.4% of samples, and below the lower control level for HAdV in one sample. Enteric viruses were detected more frequently in samples collected during the rainy seasons (1 March to 31 May and 16 October to 15 December) than the dry seasons (*p* = 0.003). Enteric viruses were detected less frequently on some sampling days, including 28 July 2015 and 11 August 2015 (*p* = 0.05) (Figure S8, available online). The eight enteric viruses were detected at similar rates between all sites (*p* = 0.05).

Of the enteric viruses detected, HRV was detected the least frequently (Table 1), though its detection rate was comparable to previous studies (Kiulia *et al*. 2015). The lower HRV detection rate compared to other enteric viruses was unexpected due to the 2014 introduction of the Rotarix^®^ vaccine in Kenya (Wandera *et al*. 2017). This live attenuated vaccine targets G1P[8] (Dóró *et al*. 2015), and is shed in stool by the inoculated population. The lower HRV detection frequency in this study could be due to less efficient HRV recovery by the BMFS compared to other enteric viruses, as these recovery rates using the BMFS are unknown. It could also indicate low vaccination coverage, though the coverage for these catchment communities is unknown. In 2015, HRV vaccine coverage in Kenya was estimated at 66% (WHO & UNICEF 2018). Finally, it may be due to the age of vaccinated individuals. As Rotarix is administered to infants at 6 and 10 weeks, vaccine strains may or may not enter the wastewater system depending on use of reusable or disposable diapers in the study area. Rotavirus is a leading cause of gastroenteritis in children <5 years and prevalence rates of 6–56% have been reported in Kenya (Kiulia *et al*. 2008). In the first year after vaccine introduction in Kenya, hospitalizations due to rotavirus gastroenteritis for children <5 years decreased by 30% and by 64% in year two, when compared to data from 2009–2014 (Wandera et al. 2017).

The enteric virus prevalence in this study is comparable with previous findings. In another Kenyan study (Kiulia *et al*. 2010), ten 1-L surface water samples from a sewagecontaminated stream in Kibera were concentrated using glass-wool adsorption-elution and PEG precipitation, then tested for the presence of eight enteric viruses using conventional nested RT-PCR. High levels of viral contamination were shown, with four or more enteric viruses detected in 100% of these samples. In contrast to the BMFS study, only EV and HRV were detected in all samples, with 60% of samples positive for HAstV. This study by Kiulia *et al*. was conducted prior to the Rotarix vaccine introduction in Kenya, which may explain the frequent detection of multiple HRV genotypes. Additionally, viruses detected in these Kenyan environmental surveillance studies have been detected in stool samples from Kibera (Shioda *et al*. 2016). Of the collected stool samples, 3% were positive for HAstV, 6% for NoV GI, 27% for NoV GII, and 6% for SaV. This suggests that viruses detected during environmental surveillance in Kibera originated from individuals living in Kibera.

The frequent detection of eight enteric viruses throughout this study in samples collected in Mombasa, Garissa, and Kisumu suggests the BMFS is a robust method that could be used in a variety of situations (Table 1). Samples were filtered in the field, shipped to Nairobi on cold chain for processing, and typically received at the Kenya Medical Research Institute (KEMRI) 1–2 days after collection. This is similar to the timeframes seen during a BMFS poliovirus environmental surveillance study conducted in Pakistan (Zhou *et al*. 2018). In Pakistan, shipping time did not affect poliovirus detection, though samples were not analyzed for other viruses. Additionally, poliovirus detection was measured as presence/absence rather than Cq value, so shipping impacts on virus concentration were not explored. Cq values for enteric viruses in samples collected outside Nairobi were similar to those collected in Nairobi, suggesting that a short shipping timeframe on cold chain may not significantly affect enteric virus survival on the filters. However, if samples may lose cold chain, preservatives could be added prior to filter shipment to further improve the likelihood of viral detection (Fagnant *et al*. 2017).

Viral contamination of surface water sources globally are well documented. An investigation into virological quality of aquatic venues during the 2016 Summer Olympic Games in Brazil showed that 95.5% of water samples were contaminated with at least one enteric virus (Staggemeier *et al*. 2017). Of the four viruses examined, EV, HAdV, and HRV were present, though no HAV was detected. Additionally, during an investigation of the virological quality of recreational water in Argentina, dam water had infectious EVs detected in 64.6% of samples, and HAstV, HAV, NoV, andHRV were detected in 22.9–60.5% of samples (Masachessi *et al*. 2018). Recently, a study performed in an urban river in Germany showed HAdV as the predominant enteric virus, followed by EV, HRV and NoV GII (Mackowiak *et al*. 2018). A review of studies around Africa showed detection of human NoV in 62.2% of water samples tested (Kabue *et al*. 2016). Also, surface water sampling in South Africa resulted in detection of EV, HAV, and HRV (Chigor & Okoh 2012). Therefore, irrespective of the socio-economic status of the country, enteric virus contamination of surface water sources is a global phenomenon, which emphasizes the need for continued environmental surveillance.

#### PMMoV detection and quantification

**Figure 2:**
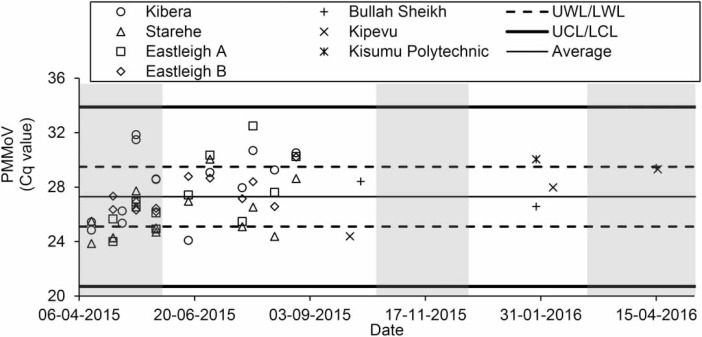
Detection of pepper mild mottle virus (PMMoV). Shaded areas show the rainy season. UWL and LWL are the upper and lower warning levels (average ± one standard deviation). UCL and LCL are the upper and lower control levels (average ± three standard deviation).

PMMoV was detected in 100% of BMFS samples and cooccurred with the presence of human enteric viruses (*n* = 59; Table 1). PMMoV was detected with similar Cq values as EV (Tables 2 and 3) and PMMoV concentrations were within the upper and lower warning levels for 62.7% of samples (Figure 2). Unlike the presence/absence trends of other enteric viruses measured (Figure S8), PMMoV was detected with significantly higher concentrations in samples collected on 29 September 2015 than 30 June 2015 or 25 August 2015 (p< 0.05). There was no statistically significant difference in PMMoV detection between the seven sites sampled (p > 0.05). The average estimated PMMoV concentration was 6.1 × 10^5^ gene copies L^1^ sample (Table 3). This is within the range seen for contaminated surface waters (10^1^–10^8^ targets L^1^), though slightly lower than the range for untreated domestic wastewater (10^6^–10^10^ targets L^-1^) (Symonds *et al*. 2018).

Sites sampled in this study were selected for poliovirus environmental surveillance by the WHO Global Polio Eradication Initiative, Kenya Ministry of Health, and KEMRI. Consistent EV and PMMoV detection at these sites indicates appropriate site selection, and suggests that EV or PMMoV could be used as a BMFS process control for enteric virus environmental surveillance at similarly contaminated sites. Additionally, by comparing the EV or PMMoV departure from baseline with the departure from baseline for other pathogenic viruses, this may indicate if exceeding the upper control level is due to a large influx of waste or of a specific virus. While EV and the other enteric viruses used the same extraction process, extraction instrument, and qPCR instrument, PMMoV RNA extraction was conducted separately in another laboratory after sample shipment from South Africa to the United States. This could lead to viral loss, though PMMoV was consistently detected at high concentrations (Table 3). To ensure sample integrity in future studies, the site indicators/process controls and target viruses should use the same nucleic acid extractions and be processed at the same location.

### CONCLUSIONS

Since surface water contamination with enteric viruses occurs in both developing and developed countries, continued enteric virus environmental surveillance is needed to detect silently circulating viruses, assist with determining a community’s disease burden, and guide vaccine efforts. This study demonstrated that the BMFS is applicable for environmental sampling for a diverse array of enteric viruses. Consistent detection of EVs and PMMoV suggests that they could be used as process controls for sampling site suitability at sites with similarly high levels of fecal pollution and the BMFS process. The variability in Cq values at some sampling sites could be due to seasonality, population dynamics, and/or disease patterns in surrounding communities. Future work should determine BMFS recovery rate efficiencies for different enteric viruses and if modifications are needed to improve detection. This study provides an efficient way to sample and concentrate large volumes for simultaneous detection of multiple enteric viruses.
