# Supplementary material for: Detection of potentially pathogenic enteric viruses in environmental samples from Kenya using the bag-mediated filtration system
Source: Water Sci Technol Water Supply. 2019 Mar 12;19(6):1668–76. doi: 10.2166/ws.2019.046 (PMC7797634; doi:10.2166/ws.2019.046)
Supplement: Supplementary file 1 [file WS-19-06-1668-s001.docx]

SUPPLEMENTARY MATERIAL

*Detection of potentially pathogenic enteric viruses in environmental samples from Kenya using the bag-mediated filtration system*

Walda B. van Zyl^1^, Nicolette A. Zhou^2^, Marianne Wolfaardt^1^, Peter N. Matsapola^1^, Fhatuwani B. Ngwana^1^, Erin M. Symonds^3^, Christine S. Fagnant-Sperati^2^, Jeffry H. Shirai^2^, Alexandra L. Kossik^2^, Nicola K. Beck^2^, Evans Komen^4^, Benlick Mwangi^4^, James Nyangao^4^, David S. Boyle^5^, Peter Borus^4^, Maureen B. Taylor^1^, J. Scott Meschke^2^*

^1^ Department of Medical Virology, University of Pretoria; Faculty of Health Sciences, Private Bag X323, Arcadia 0007 South Africa

^2^ Department of Environmental and Occupational Health Sciences, University of Washington; 4225 Roosevelt Way NE, Suite 100, Seattle, WA 98105, USA

^3^ College of Marine Science, University of South Florida; 830 1^st^ St S, St. Petersburg, FL 33701, USA

^4^ Centre for Viral Research, Kenya Medical Research Institute; Mbagathi Road, P.O. Box 54628, Nairobi 00200, Kenya

^5^ PATH; 2201 Westlake Ave, Suite 200, Seattle WA 98121, USA

*corresponding author: email: [jmeschke@uw.edu](mailto:jmeschke@uw.edu)

**Table of Contents**

[**Methods** 3](#_Toc534899999)

[**Table S1.** Primers and probes used for detection of enteric viruses 5](#_Toc534900000)

[**Table S2.** Real-time RT-PCR results, reported as the quantification cycle, from the direct analysis of sequentially collected BMFS samples 6](#_Toc534900001)

[**Figure S1.** Detection of human adenovirus (HAdV) at the seven sites sampled in Kenya. 7](#_Toc534900002)

[**Figure S2.** Detection of human astrovirus (HAstV) at the seven sites sampled in Kenya. 7](#_Toc534900003)

[**Figure S3.** Detection of hepatitis A virus (HAV) at the seven sites sampled in Kenya 8](#_Toc534900004)

[**Figure S4.** Detection of norovirus GI (NoV GI) at the seven sites sampled in Kenya. 8](#_Toc534900006)

[**Figure S5.** Detection of norovirus GII (NoV GII) at the seven sites sampled in Kenya. 9](#_Toc534900008)

[**Figure S6.** Detection of sapovirus (SaV) at the seven sites sampled in Kenya. 9](#_Toc534900009)

[**Figure S7.** Detection of human rotavirus (HRV) at the seven sites sampled in Kenya. 10](#_Toc534900011)

[**Figure S8.** Statistical significance of human enteric virus detection between sampling days. 11](#_Toc534900012)

# **Methods**

*Study permissions*

This study was approved by the Kenya Medical Research Institute (KEMRI) Scientific and Ethics Review Unit (Non-SSC Protocol #464, KEMRI/RES/7/3/1), National Commission for Science, Technology, and Innovation (NACOSTI/P/15/2731/4929), and Research Ethics Committee, Faculty of Health Sciences, University of Pretoria (UP) (Ethics Reference #119/2017). Ethics approval was also received by PATH (RDC-0772) and the University of Washington (UW) (HSD Study #48465) Institutional Review Boards/Human Subject Committees.

*Study design*

Sampling sites included four sites in Nairobi: the Motoine River bordering the Kibera informal settlement (Kibera), a sewer conveyance line outlet connecting to the Mathare River (Starehe), and sewer conveyance lines in the Eastleigh neighborhood (Eastleigh A and B). Samples were also obtained from a pumping station in Mombasa (Kipevu), and sewer conveyance lines in Garissa (Bullah Sheikh) and Kisumu (Kisumu Polytechnic).

*Sample collection and processing*

Samples were collected in a collection bag with pre-screen mesh (249-µm pore size). Samples were filtered onsite immediately or stored (4ºC, <24 hours) until filtration at a separate, secure location utilizing the previously described “bucket” modification (Zhou et al., 2018). To filter, the collection bag was hung on a tripod stand, a positively charged ViroCap™ filter (Scientific Methods, Granger, IN, USA) was attached to the bag outlet port, and the sample was filtered by gravity. The filter was transported on cold packs to KEMRI in Nairobi, where a preservative mixture (2% sodium benzoate [Alfa Aesar, Ward Hill, MA, USA] and 0.2% calcium propionate [TCI America, Portland, OR, USA]) was added, held for 20 minutes, and recovered through the filter outlet (Fagnant et al., 2017).

Viruses were eluted from filters via a single 30-minute elution with a 1.5% beef extract (BBL^TM^ Beef Extract powder; Becton, Dickinson and Company, Sparks, MD, USA) and 0.05 M glycine (Merck, Darmstadt, Germany) solution, pH 9.5. Samples were secondary concentrated using polyethylene glycol (PEG) 8000 (AMRESCO, Solon, OH, USA) precipitation and resuspended in 10-mL phosphate-buffered saline.

*RT-PCR*

At UP, samples were analyzed for EV, HAstV, HAV, NoV GI, NoV GII, and HRV by direct real-time RT-PCR using CeeramTools® kits (bioMérieux), which contain positive, negative, and internal PCR inhibition controls. Primers and probes for HAdV and SaV analyses were synthesized by Applied Biosystems (Thermo Fisher Scientific Inc., Waltham, MA, USA) (Table S1). HAdV-2 (ATCC VR 846) and SaV from a clinical specimen (Murray et al., 2013) were used as positive controls for the respective in-house real-time PCR assays, and nuclease-free water (Promega Corp., Madison, WI, USA) as negative controls. Real-time RT-PCR analyses were performed using a Lightcycler 2.0 (Roche Diagnostics, Mannheim, Germany). If inhibition was observed, a 1:10 nucleic acid dilution was re-analyzed and these Cq values were not reported.

Primers for PMMoV analysis were synthesized by Integrated DNA Technologies (Coralville, IA, USA) and probes by Applied Biosystems (TaqMan^TM^ MGB Probe, Thermo Fisher Scientific Inc.) (Table S1). RT was completed using SuperScript^TM^ IV VILO^TM^ Master Mix (Thermo Fisher Scientific Inc.), and qPCR using an ABI StepOnePlus^TM^ Real-Time PCR System (Thermo Fisher Scientific Inc.) with TaqMan® Environmental Master Mix 2.0 no UNG (Thermo Fisher Scientific Inc.). Nuclease-free water (IBI Scientific, Dubuque, IA, USA) was used as negative controls. Standard curves were determined via analysis of a dilution series in duplicate of a purified recombinant plasmid containing the PMMoV assay amplicon. qPCR inhibition was not observed when samples were diluted 1:10. To determine the average PMMoV gene copies per reaction, linear regression analysis of the duplicate Cq and gene copy numbers was executed, and average PMMoV gene copy numbers were divided by two, accounting for differences in nucleic acid type (PMMoV gene target is single-stranded, while the purified recombinant plasmid is double-stranded [Bustin et al., 2009]).

# **Table S1.** Primers and probes used for detection of enteric viruses

| Assay target | Sequence (5' to 3') | | Citation |
| --- | --- | --- | --- |
| Human adenovirus | Forward | GCC ACG GTG GGG TTT CTA AAC TT | Heim et al., 2003 |
|  | Reverse | GCC CCA GTG GTC TTA CAT GCA CAT C | Heim et al., 2003 |
|  | Probe (dye) | [FAM]-TGC ACC AGA CCC GGG CTC AGG TAC TCC GA-[TAMRA] | Heim et al., 2003 |
| Sapovirus | Forward1 | GAC CAG GCT CTC GCY ACC TAC | Chan et al., 2006 |
|  | Forward2 | TTG GCC CTC GCC ACC TAC | Chan et al., 2006 |
|  | Reverse | CCC TCC ATY TCA AAC ACT AWT TTG | Chan et al., 2006 |
|  | Probe (dye) | [FAM]-TGG TTY ATA GGY GGT AC-[MGB-NFQ] | Chan et al., 2006 |
| Pepper mild mottle virus | Forward | GAG TGG TTT GAC CTT AAC GTT TGA | Haramoto et al., 2013 |
|  | Reverse | TTG TCG GTT GCA ATG CAA GT | Zhang et al., 2006 |
|  | Probe (dye) | [FAM]-CCT ACC GAA GCA AAT G-[MGB-NFQ] | Zhang et al., 2006 |

# **Table S2.** Real-time RT-PCR results, reported as the quantification cycle, from the direct analysis of sequentially collected BMFS samples

| Date  (dd/mm/yyyy) | Site | EV | | HAdV | | HAstV | | HAV | | NoV GI | | NoV GII | | SaV | | HRV | | PMMoV | |
| --- | --- | --- | --- | --- | --- | --- | --- | --- | --- | --- | --- | --- | --- | --- | --- | --- | --- | --- | --- |
| 14/04/2015 | Kibera | 24.2 | 23.9 | 26.0 | 26.3 | 26.6 | 25.0 | 27.6 | 25.6 | 28.9 | 28.6 | 30.9 | 29.6 | 28.8 | 28.8 | 22.8 | 23.0 | 25.4 | 24.8 |
| 14/04/2015 | Starehe | 23.2 | 23.8 | 26.7 | 26.6 | 27.4 | 29.6 | 25.5 | 23.7 | 30.5 | 30.8 | 31.6 | 30.0 | 29.1 | 30.6 | 26.5 | 26.9 | 25.5 | 23.9 |
| 28/04/2015 | Starehe | 25.3 | 25.1 | 28.2 | 27.8 | 30.7 | 28.4 | 29.2 | 28.3 | 30.4 | 30.2 | 31.5 | 32.0 | 27.4 | 29.8 | 25.8 | 25.2 | 24.3 | 24.3 |
| 28/04/2015 | Eastleigh A | 25.2 | 24.6 | 25.8 | 20.2 | 35.0 | 36.0 | 32.4 | 32.5 | 29.2 | 32.4 | 32.0 | 32.6 | 33.5 | 32.7 | 32.1 | 32.1 | 25.7 | 24.0 |
| 28/04/2015 | Eastleigh B | 25.9 | 26.1 | 32.8 | 32.1 | 30.5 | 35.6 | 32.9 | 33.8 | 31.3 | 29.6 | 30.3 | 32.0 | 33.2 | 32.7 | 29.5 | 28.1 | 26.4 | 27.3 |
| 04/05/2015 | Kibera | 23.9 | 24.3 | 27.8 | 27.4 | 24.4 | 24.6 | 28.9 | 29.6 | 27.6 | 28.0 | 28.8 | 29.7 | 28.6 | 28.8 | 24.8 | 24.2 | 25.3 | 26.2 |
| 13/05/2015 | Kibera | 30.0 | 29.7 | 33.7 | 33.8 | 34.4 | 35.3 | 34.5 | 35.3 | 34.1 | 33.9 | 32.1 | 32.7 | 38.4 | 38.5 | 26.6 | 26.7 | 31.5 | 31.8 |
| 13/05/2015 | Starehe | 28.0 | 26.6 | 29.9 | 27.9 | 33.6 | 31.5 | 29.9 | 29.0 | 37.5 | 32.2 | 35.6 | 31.2 | 33.0 | 31.3 | 31.6 | 28.5 | 27.7 | 26.5 |
| 13/05/2015 | Eastleigh A | 26.4 | 27.7 | 31.8 | 31.3 | 32.6 | 30.7 | 35.9 | 34.4 | 29.5 | 30.3 | 26.6 | 26.8 | 31.0 | 31.6 | 29.8 | 29.2 | 26.5 | 26.9 |
| 13/05/2015 | Eastleigh B | 24.7 | 25.7 | 33.3 | 33.1 | 34.6 | n.d. | 27.3 | 27.0 | 32.4 | 34.5 | 30.2 | 31.5 | 31.9 | 33.8 | 28.7 | 33.2 | 26.9 | 26.3 |
| 26/05/2015 | Kibera | 26.3 | 26.7 | 32.2 | 32.2 | 30.1 | 30.5 | 31.0 | 30.4 | 35.5 | 33.6 | 33.8 | 32.8 | 31.3 | 31.6 | 26.8 | 25.7 | 28.6 | 28.6 |
| 26/05/2015 | Starehe | 25.5 | 26.7 | 28.8 | 29.1 | 31.3 | 31.2 | 26.6 | 26.8 | 31.2 | 32.1 | 28.0 | 29.8 | 30.8 | 31.4 | 30.1 | 29.6 | 24.7 | 25.0 |
| 26/05/2015 | Eastleigh A | 25.7 | 26.1 | 26.1 | 24.0 | 30.9 | 34.3 | 28.5 | 28.1 | 31.7 | 33.0 | 31.3 | 32.1 | 29.5 | 28.6 | 31.2 | 31.3 | 24.9 | 26.1 |
| 26/05/2015 | Eastleigh B | 23.6 | 23.5 | 32.0 | 32.2 | 27.3 | 26.2 | 31.8 | 32.8 | 31.2 | 32.6 | 28.9 | 27.6 | 31.9 | 31.9 | 30.8 | 28.1 | 26.4 | 26.2 |

RT-PCR, reverse transcription polymerase chain reaction; BMFS, bag-mediated filtration system; EV, enterovirus; HAdV, human adenovirus; HAstV, human astrovirus; HAV, hepatitis A virus; NoV GI, norovirus GI; NoV GII, norovirus GII; SaV, sapovirus; HRV, human rotavirus; PMMoV, pepper mild mottle virus; n.d., not detected

# **Figure S1.** Detection of human adenovirus (HAdV) at the seven sites sampled in Kenya. Shaded areas show the rainy season. UWL and LWL are the upper and lower warning levels (average ± one standard deviation). UCL and LCL are the upper and lower control levels (average ± three standard deviations).

# **Figure S2.** Detection of human astrovirus (HAstV) at the seven sites sampled in Kenya. Shaded areas show the rainy season. UWL and LWL are the upper and lower warning levels (average ± one standard deviation). UCL and LCL are the upper and lower control levels (average ± three standard deviations).

# **Figure S3.** Detection of hepatitis A virus (HAV) at the seven sites sampled in Kenya. Shaded areas show the rainy season. UWL and LWL are the upper and lower warning levels (average ± one standard deviation). UCL and LCL are the upper and lower control levels (average ± three standard deviations).

#

# **Figure S4.** Detection of norovirus GI (NoV GI) at the seven sites sampled in Kenya. Shaded areas show the rainy season. UWL and LWL are the upper and lower warning levels (average ± one standard deviation). UCL and LCL are the upper and lower control levels (average ± three standard deviations).

#

# **Figure S5.** Detection of norovirus GII (NoV GII) at the seven sites sampled in Kenya. Shaded areas show the rainy season. UWL and LWL are the upper and lower warning levels (average ± one standard deviation). UCL and LCL are the upper and lower control levels (average ± three standard deviations).

# **Figure S6.** Detection of sapovirus (SaV) at the seven sites sampled in Kenya. Shaded areas show the rainy season. UWL and LWL are the upper and lower warning levels (average ± one standard deviation). UCL and LCL are the upper and lower control levels (average ± three standard deviations).

#

# **Figure S7.** Detection of human rotavirus (HRV) at the seven sites sampled in Kenya. Shaded areas show the rainy season. UWL and LWL are the upper and lower warning levels (average ± one standard deviation). UCL and LCL are the upper and lower control levels (average ± three standard deviations).


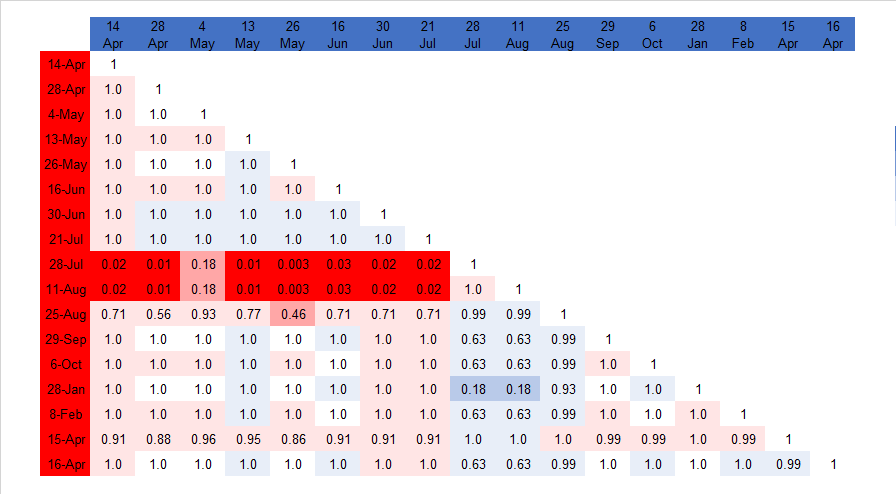


**Figure S8.** Statistical significance of human enteric virus detection between sampling days. Blue shading indicates less frequent detection on the sampling day provided in the column, and red shading indicates less frequent detection on the sampling day provided in the row.

**References**

Bustin, S. A., Benes, V., Garson, J. A., Hellemans, J., Huggett, J., Kubista, M., Mueller, R., Nolan, T., Pfaffl, M. W., Shipley, G. L., Vandesompele, J. & Wittwer, C. T. (2009) The MIQE guidelines: minimum information for publication of quantitative real-time PCR experiments. *Clinical Chemistry* **55**(4), 611–622.

Chan, M. C. W., Sung, J. J. Y., Lam, R. K. Y., Chan, P. K. S., Lai, R. W. M. & Leung, W. K. (2006) Sapovirus detection by quantitative real-time RT-PCR in clinical stool specimens. Journal of *Virological Methods* **134**(1–2), 146–153.

Fagnant, C. S., Kossik, A. L., Zhou, N. A., Sánchez-Gonzalez, L., Falman, J. C., Keim, E. K., Linden, Y., Scheibe, A., Barnes, K. S., Beck, N. K., Boyle, D. S. & Meschke, J. S. (2017) Use of preservative agents and antibiotics for increased poliovirus survival on positively charged filters. *Food and Environmental Virology* **9**(4), 383–394.

Haramoto, E., Kitajima, M., Kishida, N., Konno, Y., Katayama, H., Asami, M. & Akiba, M. (2013) Occurrence of pepper mild mottle virus in drinking water sources in Japan. A*pplied and Environmental Microbiology* **79**(23), 7413–7418.

Heim, A., Ebnet, C., Harste, G. & Pring-Akerblom, P. (2003) Rapid and quantitative detection of human adenovirus DNA by real-time PCR. J*ournal of Medical Virology* **70**(2), 228–239.

Murray, T. Y., Mans, J. & Taylor, M. B. (2013) Human calicivirus diversity in wastewater in South Africa. *Journal of Applied Microbiology* **114**(6), 1843–1853.

Zhang, T., Breitbart, M., Lee, W. H., Run, J.-Q., Wei, C. L., Soh, S. W. L., Hibberd, M. L., Liu, E. T., Rohwer, F. & Ruan, Y. (2006) RNA Viral Community in Human Feces: Prevalence of Plant Pathogenic Viruses. *PLOS Biology* **4**(1), e3.

Zhou, N. A., Fagnant-Sperati, C. S., Shirai, J. H., Sharif, S., Zaidi, S. Z., Rehman, L., Hussain, J., Agha, R., Shaukat, S., Alam, M., Khurshid, A., Mujtaba, G., Salman, M., Safdar, R. M., Mahamud, A., Ahmed, J., Khan, S., Kossik, A. L., Beck, N. K., Matrajt, G., Asghar, H., Bandyopadhyay, A. S., Boyle, D. S. & Meschke, J. S. (2018) Evaluation of the bag-mediated filtration system as a novel tool for poliovirus environmental surveillance: Results from a comparative field study in Pakistan. *PLOS ONE* **13**(7), e0200551.
